# Dental Education

**Published:** 1866-10

**Authors:** 


### DENTAL EDUCATION.

This may seem to be a hackneyed subject, but it requires a strong agitation nevertheless, and it is full time a move was made in the matter, and that the agitation consist of something more than mere words.

The necessity for such a movement is apparent to and acknowledged by almost every one in the profession; and it is certainly a matter of great interest not only to the profession, but to all. The call for reformation in this matter of medical education is loud and clear, and particularly is this true in reference to our own specialty. Many resolve and seek to enter the ranks of the dental profession with scarcely any conception of the attainments requisite to constitute a reputable and efficient practitioner of dentistry; some care nothing for it, further than it may be a means of making money.

There are always some who will be influenced by selfish motives. There are others who from a want of proper conception, having started in the wrong way, find it exceedingly difficult to get into the right. And hence the great importance of *starting right.* Some have been disposed to find fault with our Dental Colleges and those who have in some sort served as educators in our profession; but the fault does not begin here, but it has its beginning with those members of the profession who fail to impress upon the mind of the young man contemplating a course of study a proper conception of its character and extent. Too often he is led to believe that it is but the work of a few months to obtain all that is necessary to constitute an efficient and successful practitioner; that it consists simply in attaining some mechanical and manipulative ability, together with a little smattering of general principles, while general literary and scientific education is wholly overlooked.

